# Evolving trends in mAb production processes

**DOI:** 10.1002/btm2.10061

**Published:** 2017-04-03

**Authors:** Abhinav A. Shukla, Leslie S. Wolfe, Sigma S. Mostafa, Carnley Norman

**Affiliations:** ^1^ Process Development & Manufacturing KBI Biopharma Inc. Durham NC 27704

**Keywords:** biosimilars, bispecific antibodies, continuous bioprocessing, Fc fusion proteins, high titer cell culture, monoclonal antibodies, next generation monoclonal antibodies, nonchromatographic separations, platform process, process development

## Abstract

Monoclonal antibodies (mAbs) have established themselves as the leading biopharmaceutical therapeutic modality. The establishment of robust manufacturing platforms are key for antibody drug discovery efforts to seamlessly translate into clinical and commercial successes. Several drivers are influencing the design of mAb manufacturing processes. The advent of biosimilars is driving a desire to achieve lower cost of goods and globalize biologics manufacturing. High titers are now routinely achieved for mAbs in mammalian cell culture. These drivers have resulted in significant evolution in process platform approaches. Additionally, several new trends in bioprocessing have arisen in keeping with these needs. These include the consideration of alternative expression systems, continuous biomanufacturing and non‐chromatographic separation formats. This paper discusses these drivers in the context of the kinds of changes they are driving in mAb production processes.

## 1 Introduction to mAb platform processes

Monoclonal antibodies (mAbs) are the most successful class of biopharmaceuticals today. More than 50 mAbs have been approved and sales of mAbs are expected to cross $125 billion by 2020.^1^ The ability to bind to specific targets with high specificity and affinity and the ease of developing human or humanized sequences to a target have been behind the explosive growth of this class of pharmaceutical products. The last 20 years have seen the rapid growth of this class of therapeutics with over 300 mAbs in clinical development today. Today mAbs are approved for a wide range of indications covering oncology,^2^ autoimmune disorders and rare disease indications.

While the ability to target cell surface targets with great specificity have been behind the rise of mAbs,^3^ a key enabler has been the ability to rapidly develop robust manufacturing processes that can bring mAb product candidates into clinical trials. The ease and speed of producing mAbs has enabled rapid entry of these product candidates into clinical trials and the scalability and robustness of these processes has hugely facilitated large scale commercial supply.^4^, ^5^

Development of a manufacturing process for a protein requires the consideration of many different factors including removal of impurities, robustness, scalability, and ready availability of raw materials for large‐scale production. Consideration has to be given not only to the scale needed for early clinical supply, but also the ability of the process to support long‐term supply needs and scales. As a result, utilizing well‐established unit operations is a key aspect of developing manufacturing processes. The aspects of robustness, scalability, and reproducibility mean that manufacturing processes often look quite different from those that can be employed in the laboratory for purifying small quantities of proteins. Process development can be a time consuming activity and require significant amounts of experimentation. As a result, when possible the industry has gravitated toward platform approaches.

A platform approach has distinct advantages from a business standpoint. Speed to clinic is often a key determinant of a company's success. mAb platforms have enabled progression from gene to IND in less than a year, which is a significant improvement over molecules that require involved development efforts that can extend up to 2 years. This reduced experimentation also implies a reduction in the cost of the development effort. The predictability of a process platform enables organizations such as Manufacturing and Quality Control to adopt a templated set of documents which also reduces the time and resources spent on production and release testing. mAb process platforms have enabled a highly productive, robust manufacturing process to be put in place from the start of clinical development all the way to product commercialization. The consistency and predictability of a platform approach have significantly enabled the growth of this class of therapeutics.

mAb therapeutics particularly lend themselves toward the application of platform approaches. Use of a well‐developed mammalian cell culture expression system enables development of stable cell lines in a very rapid and templated fashion for mAbs. Several expression vectors have been optimized specifically for mAb production.^6^, ^7^ Robust fed‐batch cell culture processes have been developed for mAbs. Several of these have been scaled to large‐scale production and characterized extensively giving a good idea of the operating parameters that influence these processes.^8^, ^9^ Cell line development and upstream cell culture processes lend themselves to a templated approach very well. However, for most proteins the greatest area of divergence comes in the form of the downstream purification process that has to be customized for each protein based on its properties as well as that of key impurities. The Fc region of mAbs binds very specifically to immobilized Protein A which is a cell wall component of *Staphylococcus aureus*. Protein A affinity chromatography has been shown to be widely applicable for mAbs and can achieve >95% purity with very little development on this mode of chromatography.^10^ The chief challenge after Protein A chromatography is to remove residual host cell protein impurities, high molecular weight aggregate, DNA, and have the capability of removing adventitious viruses. A number of downstream process platforms for mAbs have been developed at leading biopharmaceutical companies.^4^, ^5^, ^11^, ^12^, ^13^ The ability to use a generic approach across molecules and starting from a template decrease the amount of experimentation needed compared with a protein that cannot enable the inclusion of an affinity step in the downstream process.

These downstream process platforms have successfully enabled the progression of a large number of mAb products into the clinic and commercial space. However, several emerging trends are continuing to shape the biopharmaceutical industry today. These trends are discussed in the next section in the context of their being drivers for changes in what mAb production processes look like today.

## 2 Forces driving changes in biopharmaceuticals

A number of factors are driving a modification in traditional biopharmaceutical manufacturing in which production cost was not considered an important factor since the selling price of the drug was dictated by value added to patient life and health. As a result, cost pf production is coming increasingly into focus. Additionally, organizations with manufacturing capacity are looking to make more efficient use of existing plants to reduce the need for new plant construction. A number of these factors have been examined in the context of driving process innovation.^14^, ^15^

### 2.1 Biosimilars

The advent of follow‐on biologics (a.k.a biosimilars in popular parlance) is a key change to traditional biopharmaceutical paradigms.^16^, ^17^ Even though it does not appear that the price decrease in biopharmaceuticals is as significant as for small molecule drugs due to their complexity, nevertheless follow‐on biologics are driving a focus on cost‐conscious manufacturing. The European Union enacted the “Guideline on similar biological medicinal products” on October 30, 2005. A number of big biotech and big pharma companies have since then announced the formation of biosimilars initiatives including Sandoz, Amgen, Biogen (along with Samsung), Pfizer and Merck & Co. Estimates of market size for biosimilars is close to $20 billion by 2020. The U.S. FDA has been more cautious in accepting biosimilar applications, but has approved two products till date Zarxio (Neupogen biosimilar) and Inflectra (Remicade biosimilar). Conversely, the EMA had approved 22 biosimilars till May 2016. As this trend grows worldwide, it will drive a strong interest in reducing cost of goods (COGS).

### 2.2 Globalized biomanufacturing

Along with the rise of biosimilars, there is a growth of interest in globalized biomanufacturing. This has to do with several markets giving preferential treatment to manufacturers that produce their biopharmaceuticals locally. This is particularly true of China where Pfizer and GE have teamed up to launch a biosimilars manufacturing plant called KU Bio. Other biomanufacturing players have also entered the Chinese market including WuXi. In December 2015, the Chinese FDA (cFDA) has announced fast track marketing approvals for companies producing their products in China. Similar local biomanufacturing trends are starting to gather steam in Latin America and South Africa. The list of Indian companies engaged in biopharmaceutical manufacturing has also grown substantially.

### 2.3 Single‐use manufacturing technologies

Globalized manufacturing is enabled by the growth in single‐use manufacturing technologies that require significantly lower capital investment to construct. Single‐use manufacturing saw rapid adoption for clinical manufacturing as end‐to‐end production using disposable technologies became possible.^18^, ^19^ Single‐use manufacturing of biopharmaceuticals is a trend that has now extended itself to commercial manufacturing as well, with several manufacturers utilizing multiple 2000L single‐use bioreactors in tandem to produce biopharmaceuticals at significant scale. A case in point is Amgen's manufacturing facility in Singapore that utilized 6 × 2000L single‐use bioreactors for cell culture production. These technologies are making cGMP manufacturing of biopharmaceutical significantly more accessible than the large stainless steel facilities that have dominated the industry till date. When combined with a modular construction that can be assembled together rapidly, this becomes a technology that can expand biomanufacturing worldwide.

### 2.4 Increase in cell culture titers

Product expression in cell culture bioreactors has also increased quite significantly from a few decades ago. Today, mAbs can routinely be expressed at titers of > 5 g/L in 14 day fed‐batch production. Continuous culture via perfusion bioreactors can expand reactor productivity even more significantly. These changes have been enabled by advances in cell line expression vectors, clone selection as well as in cell culture media. This rise in product expression in turn enables the use of smaller scale bioreactors (such as 2000L single‐use bioreactors) for commercial production.

### 2.5 New downstream process technologies

The increase of upstream process productivity has placed the production bottleneck in downstream processing. Even though it has been argued that current downstream technologies using fixed chromatographic column formats can meet the demands of multi‐ton production of mAbs,^4^, ^20^ there is nevertheless increased interest in purification technologies that can significantly boost productivity. This includes a recent interest in continuous bioprocessing (often mentioned in an integrated sense between continuous upstream perfusion and downstream operation) as well as a driver toward nonchromatographic techniques that could be employed at large‐scale.^21^ Another area of renewed interest is that of nonchromatographic separations in which the dependence on chromatographic columns that are intrinsically throughput limited is reduced.

### 2.6 Next generation antibody constructs

The other major factor that leads to evolution of the platform approach is the specific mAb‐like construct that is being developed as a potential therapeutic. The biotechnology industry is rapidly moving beyond conventional mAbs into a variety of constructs including Fc fusion proteins, bispecific antibodies (bsAbs) and antibody‐fusion proteins. Each of these new constructs require modifications of the original mAb platform process to enable their production.

Fc fusion proteins are created by joining the coding sequence for the Fc region of a mAb to the coding sequence for another protein.^22^ The Fc region offers several advantages as a fusion partner. Many biologically active peptides and proteins have a short serum half‐life reflecting their rapid clearance through the kidneys. The Fc region can bind to the neonatal Fc receptor to extend the half‐life of antibodies, and the same benefits are conferred on fusion partners.^23^ Seven Fc fusion proteins are commercially available as approved biopharmaceuticals and at least two of them (Enbrel and Orencia) have already achieved blockbuster status with sales of over US$1 billion per year. The original platform for mAbs at Amgen included the purification of Fc fusion proteins.^5^ However, several key downstream differences do exist for Fc fusion proteins including the possibility of susceptibility to proteolytic cleavage and the possibility of higher high molecular weight aggregate (HMW) levels being present than regular mAbs. A typical mAb downstream platform approach usually is effective, with possible adjustments to the polishing steps to account for stability of the molecule and effective HMW clearance.

Bispecific antibodies (bsAbs) are designed proteins capable of simultaneously binding and neutralizing two different antigens (ligands, cell receptor, or cytokines) or two distinct epitopes on the same antigen.^24^ As a result of this property, bsAbs can serve as mediators in redirecting immune effectors and cytotoxic agents like T‐cells to tumors or to infecting organisms such as bacteria. The fact that two arms of the mAb are different leads to several processing challenges that are unique to bsAbs. For example if the two halves of the mAb are expressed separately, a downstream process that requires disassembly of the two halves followed by reassembly to form the heterodimeric species will have to be conducted.^25^ In addition to the recombination steps, even if the formation of homodimeric species is discouraged (by use of a knob‐in‐hole methodology or a similar technology that encourages heterodimer formation), small quantities of the homodimers will form and will have to be removed by downstream processing. As a result, this format of bsAbs requires a more complex downstream process.

However, use of a common light chain along with the knob‐in‐hole technology (KiH) enables the formation of a bsAb in cell culture expression.^26^ Homodimer formation is discouraged due to the KiH construct. The small quantities of homodimer that are formed can be removed if the sequences selected for those have a biochemical difference. This can be done even in the absence of a common light chain. For example in the XmAb technology from Xencor, the homodimer and heterodimer have a small difference in charge in their engineered Fc regions that enables their separation on CEX. The other purpose of the engineering approach is the enhance effector function of the bSAb.^27^

An antibody‐drug conjugate (ADC) is a mAb conjugated with a cytotoxic agent via a linker with the primary aim of treating cancer.^28^ ADCs can be produced in a downstream process identically to conventional mAbs except for a chemical conjugation step at the end of the downstream process. This is typically followed by UF/DF to remove the conjugating chemicals. The chief difference from mAbs is the requirement for more stringent containment and personnel protection due to the toxic nature of the conjugate.

**Figure 1:**
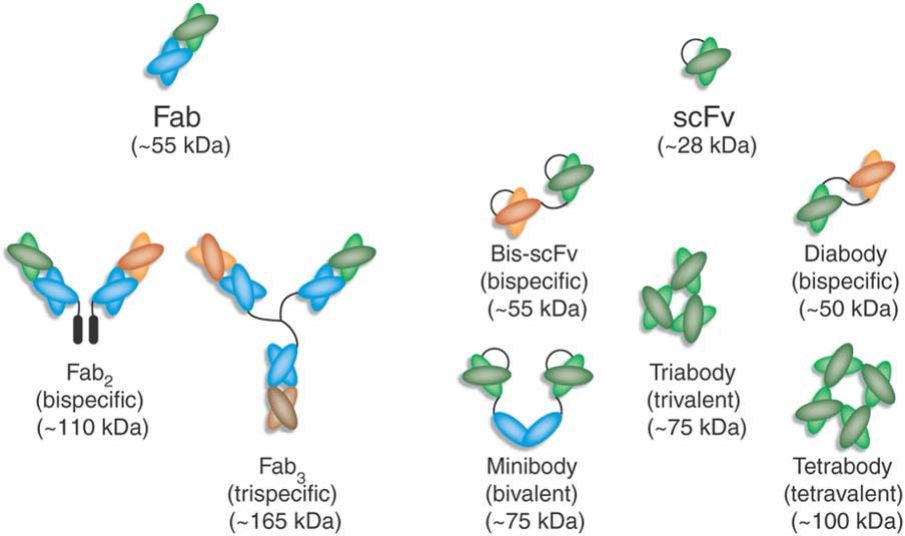
Possible next generation antibody formats

Other next generation mAb constructs include peptide fusions to mAbs at either the C or N termini to further enhance the ability to bind to more than one target at a time. Examples include anticalins fused to a mAb structure.^29^ A whole range of other therapeutic options exist as shown in Figure 1, including engineered scFvs, diabodies and tribodies, and Fab conjugates in the form of dimers or trimers.^30^ Each of these therapeutic modalities can lead to the development of platform process approaches if they become prevalent as a therapeutic modality.

## 3 Current and evolving upstream and downstream process platforms for mAbs

### 3.1 Drivers for mAb platform evolution

This section describes several downstream process schemes that have been developed at leading biopharmaceutical companies and successfully employed for mAb manufacturing at large scale. Several aspects are in common across these process schemes. mAbs are extracellularly secreted into the cell culture medium during mammalian cell culture. Harvest and recovery schemes typically utilize centrifugation followed by depth filtration and a series of membrane filters.^31^, ^32^ At smaller scales, centrifugation may be dispensed with and a series of depth filters with the coarser pore size filters first may often be utilized. The platform process almost always commences with Protein A chromatographic capture.^33^ In some cases, selective washes can be built into the Protein A step operation to further enhance host cell proteins (HCP) clearance.^34^ Non‐Protein A schemes have been developed and employed for large‐scale manufacturing^35^ as for any therapeutic protein but have not caught on given the absence of a generic approach and issues with process robustness. The process will also include two dedicated orthogonal viral clearance steps as per ICH guidelines. Low pH viral inactivation and viral filtration through a parvoviral grade filter are widely employed for mAbs.^36^ The low pH incubation step is typically placed immediately after Protein A chromatography since the Protein A column elutes in a low pH buffer. Following Protein A chromatography and viral inactivation, one or two polishing chromatographic steps are employed to clear high molecular weight aggregate,^37^ host cell proteins,^38^ DNA and to provide viral clearance potential. Chief differences among platform approaches lie in the nature of the polishing chromatographic steps. Given the high titer cell culture processes that are increasingly common in industry today (5–10 g/L), there is a significant need for high column loadings on polishing chromatographic steps.

Each company ends up customizing its mAb downstream platform approach based upon its own expression system and cell culture process as well as the mAb type and subclass they predominantly employ in discovery research. The primary criterion is that the platform approach needs to be robust and applicable across a wide range of IgG molecules without significant modification. Another key criterion is the ability of the platform to fit in the manufacturing schedule and minimize the amount of time spent in downstream processing for any one batch. As a result, another key driver is the loading capacity that is possible for any of the polishing steps.

### 3.2 Recent downstream platforms for mAbs

**Figure 2:**
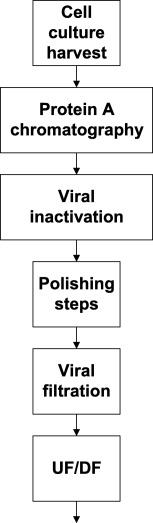
Platform approach to mAb downstream processing at Amgen

Amgen was one of the first companies to disclose its approach to a downstream process platform.^5^ A completely templated approach was shown not to be possible, but a small number of development experiments should lead to answers about process parameters that need to be customized.^11^ Examples of such process parameters include examples such as the Protein A elution pH and the choice of polishing chromatographic steps depending on the chief set of impurities that need clearance. Figure 2 shows the platform downstream scheme in use at Amgen at the time. The polishing steps typically employed were cation‐exchange chromatography (CEX) in a bind and elute mode followed by either hydrophobic interaction chromatography (HIC) in a flow‐through mode if high molecular weight aggregates needed to be cleared or anion‐exchange (AEX) chromatography in a flow through mode if only host cell protein clearance was needed. In a few cases, hydroxyapatite was employed if specific product‐related impurities needed to be removed. In situations in which adequate HCP and HMW clearance were obtained after the first polishing CEX step, AEX membrane chromatography was employed to provide for another step with viral clearance potential.^39^

**Figure 3:**
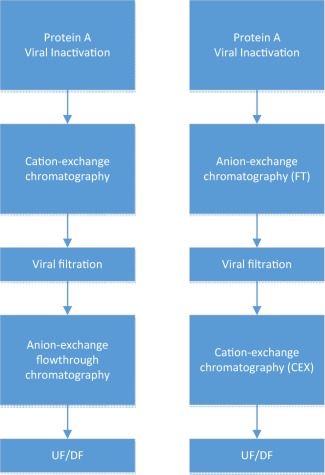
Genentech mAb downstream process platform

Another leading company to develop a pipeline of mAbs using a platform approach was Genentech.^4^, ^20^, ^40^ Genentech historically employed CEX and AEX chromatography as part of its downstream platform (Figure 3). The trend appeared to initially be to utilize CEX in a bind and elute mode as the first polishing step followed by AEX in a flow‐through mode of operation. mAbs usually have a basic pI and hence bind tightly to CEX resins and tend to flow‐through readily on AEX resins. The CEX step clears both HMW and HCP. The AEX step however is noted to require a low load conductivity to successfully remove HCPs. This can lead to a bottleneck during plant operation due to the need for large volume in‐process hold tanks to hold the AEX load. The possible use of mixed‐mode Captoadhere resin in place of more typical AEX resins is mentioned to help reduce the need for significant dilution.

One of the key drawbacks of using AEX is that it only provides clearance for HCPs and not for HMW aggregates. This particular drawback has been mitigated by the development of an AEX step operated in a weak partitioning mode^41^ in which the product actually has some measure of retention on the resin. In this mode of operation, the AEX step has actually been shown to be capable of reducing HMW content in addition to HCP species. Mechanistically, we speculate that interactions with the somewhat hydrophobic parts of the resin backbone set in at very low conductivities enabling some degree of HMW removal to occur.

**Figure 4:**
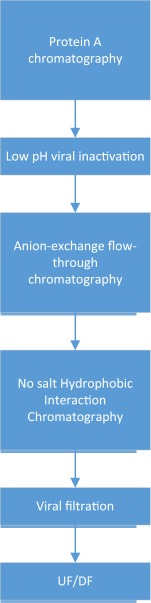
Platform mAb downstream process at Biogen

Both of the above schemes suffer from the drawback of requiring significant dilution of the load material for the AEX step. In addition, CEX and AEX alone are often unable to provide adequate HMW clearance. HMW levels can vary for mAbs but levels of > 5% HMW aggregate after Protein A chromatography are not that uncommon. Such levels often require recourse to a HIC step as has been mentioned before.^5^ Another innovative approach has been to operate the HIC step under highly overloaded conditions (>200 g/L) under no salt conditions.^42^ This approach entails the use of a highly hydrophobic HIC resin with no additional kosmotropic salt added to the load. pH conditions of the load are modulated to enable HMW removal under these extreme overloaded conditions. The use of such an ultra‐high capacity step enables the processing of large production quantities of mAbs and is used as part of the mAb platform at Biogen (Figure 4) in combination with a flow‐through AEX step. The ability to load the polishing steps at very high loads enables a reduction in column cycling and decreases the duration of floor time taken for a batch.

A similar flow‐through only polishing scheme has been discussed by Millipore‐Sigma.^43^ In this proposed format, CEX would be operated under highly overloaded conditions during the loading step. When operated in this fashion, CEX has the ability to clear some level of HCPs. It is again speculated by us that this occurs due to weak hydrophobic interactions with the chromatographic backbone. This polishing step is then combined with AEX flow‐through to complete the sequence.

**Figure 5:**
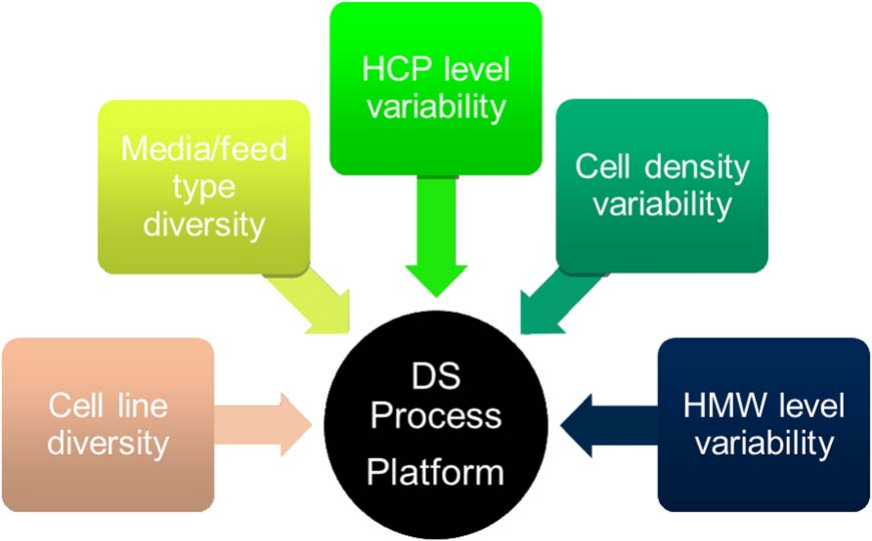
Range of factors considered in determining KBI Biopharma's platform approach

Contract development and manufacturing (CMDO) requires the ability to incorporate many different cell lines and cell culture processes into the downstream platform approach. This places significant challenges on the downstream platform scheme since both HCP and HMW reduction are simultaneously required. These simultaneous demands on the downstream mAb platform are illustrated in Figure 5. Varying cell lines and media types represent a larger degree of variability that the downstream process platform is asked to deal with. As a result, both polishing steps need to be capable of clearing HMW and HCP simultaneously.

### 3.3 A broadly applicable mAb downstream platform

**Figure 6:**
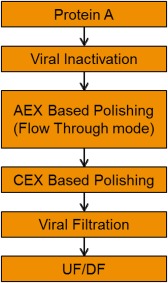
KBI Biopharma's approach to mAb downstream processing for FIH manufacturing

CEX and AEX in their traditional flavors do not fulfil this need. A key modification made in this regard has been the use of multimodal chromatography as part of the mAb downstream platform. Multimodal chromatography involves the incorporation of a hydrophobic moiety into the ligands structure for either anion‐exchange or CEX.^44^ The increased hydrophobicity of the chromatographic resin now enable improved clearance of HMW on both CEX and AEX modes which are arguably most suited for mAb processing. In addition, both these modes of chromatography are also capable of HCP and DNA clearance as well. mAbs do differ in terms of their own hydrophobicity. As a result the platform downstream process for mAbs at KBI is defined as anion‐exchange chromatography (with resin hydrophobicity that can vary from Q Sepharose FF or Capto Q to Fractogel SO3 to multimodal chromatographic resins such as Captoadhere and Nuvia cPrime). This modulation of hydrophobicity enables optimal conditions to be tailored for each mAb. Similarly, the CEX bind and elute step is also operated on a range of hydrophobicities ranging from mild to moderate depending on the resin selected. While this approach does entail some degree of experimental work, a preferred approach using multimodal chromatography for both AEX and CEX is employed as the primary platform with recourse to less hydrophobic stationary phases should the mAb require it. This approach is illustrated in Figure 6 and has been useful in terms of its breadth of covering a wide range of mAb constructs, cell lines and cell culture processes at KBI.

**Figure 7:**
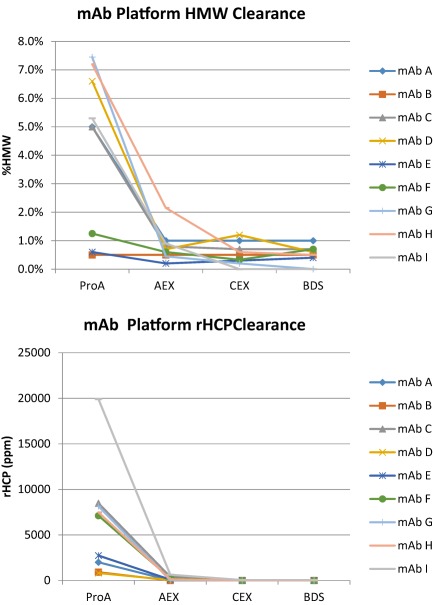
Performance of the KBI Biopharma platform DSP approach for several mAbs. (a) HMW clearance, (b) HCP clearance

Figure 7 shows the clearance profiles for HMW aggregates and HCPs through this platform process for a number of mAbs. As can be seen from the Figure, HMW aggregate levels of <1% and low HCP levels <50 ppm are always observed using this platform. The ability to cover a wide range of mAb constructs is key, especially for a CMDO such as KBI.

## 4 Emerging Process Technologies with Possible Impact on mAb Processing

The current state of the art of fed‐batch cell culture production of mAbs is dealt with reasonably well by various downstream platform processes. However, the increasing productivity in cell culture has led to the investigation of alternate methodologies to boost downstream process productivity. These technologies are seeing a significant amount of interest in the bioprocessing field due to the desire to utilize existing manufacturing facilities more fully and to the desire to reduce the COGS for biosimilar compounds. The ultimate goal will be to reduce the cost of produced biosimilars to < $10 per gram.

### 4.1 Continuous manufacturing

Small molecule pharmaceuticals have often employed continuous production methodologies to maximize productivity from manufacturing facilities. However, biopharmaceutical manufacturing has traditionally been confined to the vision of a discrete batch, all the way from the cell culture production process to downstream chromatographic steps. At this point in the field, there is growing interest in realizing the value of continuous processing for biopharmaceutical manufacturing as well.^45^ Perfusion cell culture processes in which fresh medium is added on a continuous basis to the culture and spent medium containing product is continuously withdrawn have been employed predominantly for unstable or low titer products in the past. Perfusion cell culture processes have traditionally been only employed for inhibitory or unstable products that could not be expressed to high levels in fed‐batch culture. However, more recently perfusion cell culture processes are becoming more prevalent, primarily for reasons of productivity rather than anything to do with the product itself. These processes can certainly boost productivity from a given bioreactor volume since the cells do not spend time growing from a low inoculum density but are maintained in the production phase at a high cell density via a continuous medium feed. The disadvantage of requiring large volumes of cell culture media has been dealt with logistically and the higher cost of media justified by the higher productivity achieved in such systems. Additionally, improvements in cell retention technology (e.g., the alternating tangential flow technology from Repligen) have also contributed to the more widespread adoption of perfusion cell culture at large scale.

**Figure 8:**
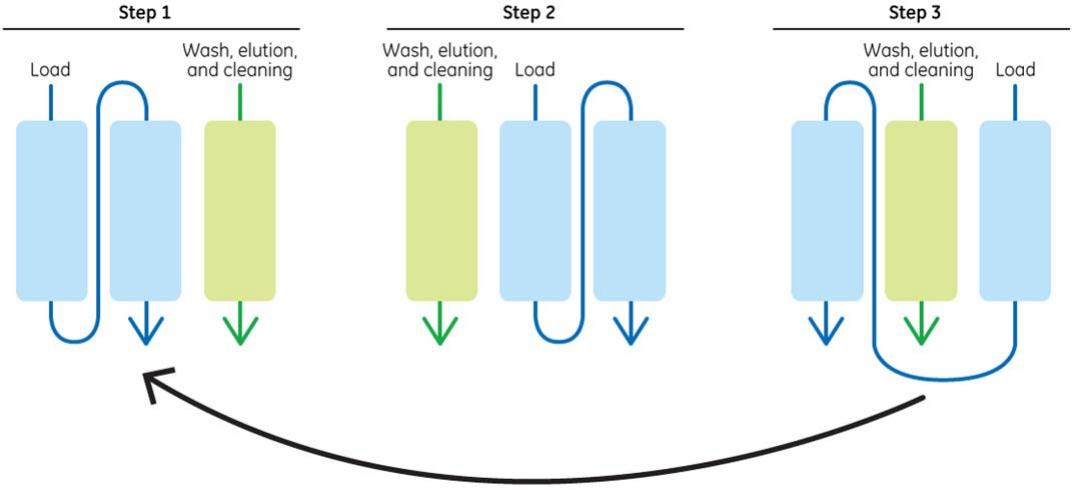
Principle of continuous chromatography

While purification of product from continuous perfusion cultures can be handled via multiple cycles on batch chromatography systems, it has also led to renewed interest in integrating continuous upstream and downstream processes. Traditional chromatography is a batch process. Steps start from column sanitization, equilibration, and loading through to washes, elution, strip and column regeneration, and storage. A batch operation is limited in terms of its throughput and productivity. First of all chromatographic columns can only be operated at a maximum diameter of 2 m owing to flow distribution limitations. Current resins can only be packed to a maximum of 30 cm bed height (20 cm typically) owing to pressure drop limitations. This inherently limits the amount of product that can be processed per cycle. When multiple cycles are required, product intermediate needs to be held for a longer duration of time. Product hold steps also require significant tank volumes in manufacturing facilities which poses another limitation to productivity. Continuous chromatographic separations can turn this batch operation into a continuous or semi‐continuous process^45^ as shown in Figure 8. Combined with a continuous upstream perfusion cell culture process, this can alter the current paradigm of how biopharmaceutical production processes are designed.^46^, ^47^ Recent economic analyses of continuous production processes indicate that continuous production techniques can achieve the same COGS from a significantly smaller bioreactor as compared to conventional batch production processes.^48^ This can significantly reduce the capital outlay required to build a manufacturing facility that is capable of commercial supply. This combines well with the drivers for more globalized production of biosimilars at low cost that was discussed in Sections 2.1 and 2.2. This analysis indicated that continuous production operations even at a 500L bioreactor scale can achieve COGS as low as $17/g.

Continuous chromatographic separation can be conducted using one of many formats. Periodic counter current chromatography is one of the formats (from GE Healthcare) and utilizes multiple columns that are continuously operated in different phases of the operation cycle.^49^, ^50^ Other formats include multicolumn countercurrent solvent gradient purification that enables recycling of the front and tail ends of the peaks to boost yield as well as purity via the ContiChrom^®^system from ChromaCon, the BioSMB™ technology from Tarpon Biosystems (now part of Pall Corporation) and the Octave chromatography system from Semba Bio.

A recent debate in the field is on how or whether end‐to‐end continuous processes are required or desired to provide the highest possible productivity.^51^ Such an objective would require the ability to operate multiple process steps in a continuous fashion including viral inactivation, ultrafiltration/diafiltration, and viral filtration steps. While technologies to conduct these in a continuous will emerge over time, it is debatable about whether a fully continuous process is necessarily needed. It may be adequate to focus on combining continuous cell culture with a continuous capture chromatographic step and then complete the rest of the mAb downstream process via high loading polishing steps described in Section 3.2.

Further progress in the use of continuous production technologies is inevitable given the number of vendors creating and marketing systems tailored for continuous processing. Key technical hurdles have been overcome in this area and the focus is now on validation of these systems for large‐scale operation as well as the use of rapid in‐process analytics for improved control of these systems. Out of the emerging technology areas listed in this paper, continuous production appears to be the first system that will see large‐scale implementation since it still leverages the selectivity offered by conventional chromatography resins.

### 4.2 Nonchromatographic separations

Another means of boosting productivity is to move from chromatographic operations to nonchromatographic separations. A key reason behind the dependence on chromatography is its ability to resolve a wide range of host cell protein impurities as well as carry out separations of species that are more closely related to the product. However, chromatographic steps are inherently throughput limited particularly when it comes to processing large batches of product which arise from operating high titer processes in large volume bioreactors. The reliance on chromatographic steps for bioseparations, limits the operation to a chromatography column of limited bed diameter and bed height. Most preparative chromatographic resins are compressible, which means they cannot be packed beyond a certain bed height and still be operated using low pressure pumps and systems currently in use in cGMP manufacturing of biopharmaceuticals. Also, the largest diameter chromatographic columns that are currently employed top out at 2 m in diameter. As a result, even with a continuous chromatography setup, throughput will still be intrinsically limited. The vision of nonchromatographic separations is that instead of employing chromatography, bioseparations could be conducted using alternative unit operations that can process an entire batch of cell culture material in one go.^52^, ^53^ This has the potential of significantly expanding the throughput of bioprocesses.

Selective precipitation schemes using polymers can be utilized to capture the entire bioreactor batch in one operation rather than relying on multiple chromatographic cycles. If these types of unit operations can be designed to be highly selective and generic in nature, they will find acceptance in large‐scale bioprocessing. A variety of polymers have been utilized for mAb precipitation including polyanionic polymers to precipitate the product^54^ and caprylic acid precipitation of host cell protein impurities.^55^ Combinations of polymers with different mechanisms can be employed to create improved selectivity. For example, salt (ionic strength driven precipitation) may be combined with charged polymers that work via charge neutralization or with PEG (polyethylene glycol) that works via exclusion of the protein molecule.^56^ Arriving at suitable combinations of agents has the potential of creating highly selective separations that can then reduce the dependency on multiple consecutive chromatography steps in the downstream process. It may also be possible to design polymers that can demonstrate multiple mechanisms and result in selective precipitation of host cell protein impurities or the product.

Another extension of selective precipitation is flocculation of the cell containing supernatant from the bioreactor.^57^ Flocculation agents such as low pH (< pH 5.0) and polymeric agents such as polydiallyldimethylammonium chloride^58^ can be used to not only precipitate cells and cell debris, but also precipitate out impurities such as host cell proteins and DNA. As such, flocculation can also be employed for impurity removal at large‐scale in addition to its role in harvest. In certain cases it can remove a substantial amount of host cell proteins such that the number of chromatographic steps in the downstream process could be possibly reduced.

Aqueous two‐phase separations (ATPS) rely upon the creation of two separate phases in solution by mixing a polymer and salt or two polymers with each other. Examples include PEG‐salt and dextran‐PEG ATPS.^59^ Several highly selective separations have been reported using ATPS. However, application at large‐scale has been limited owing to the fact that partitioning mechanisms are hard to develop and often not generic enough to enable application for a class of proteins such as mAbs. A PEG/phosphate ATPS system has been used for recovery of mAbs from transgenic plant extracts.^60^ More recently, multi‐stage ATPS have been developed in an attempt to create a single platform for many different kinds of mAbs.^61^ Further developments in ATPS using are expected in the future given the interest in this area of separations.

All of these separation technologies have interesting possibilities and can significantly raise the bar on throughput that is currently possible from manufacturing facilities. However, all of these techniques need further development to establish themselves as scalable technology that can be applied without significant development for a wide range of mAbs.

### 4.3 Alternative expression systems

mAbs can be produced in alternative expression systems in addition to mammalian cell culture. A key area of further development is the investigation of alternative expression systems that can produce mAbs with even higher productivity while preserving glycosylation patterns that are compatible with the human immune system. These developments could possibly take the field beyond the current state‐of‐the‐art production in CHO cell culture.^62^

A key alternative expression system is that of transgenic plants.^63^, ^64^ Some of these systems are already in use for clinical production such as transgenic tobacco.^65^ Transgenic plant production in tobacco is effected by transient transfection using Agrobacterium. This approach has been utilized for producing broadly neutralizing antibodies against HIV.^66^ A number of companies have emerged that employ tobacco as the expression system of choice (Medicago, Kentucky Bioprocessing). However, challenges with transgenic plant expression remain including the presence of high levels of endotoxin and the relatively low expression levels. Another concern is the secretion of proteases that lead to limited shelf life for the plant extracts. Additionally, concerns over transgenic plant growth currently limit the scalability of this technology for producing very large scale production that will be needed for commercial supply. The requirement for segregating transgenic plants from the general ecosystem means that their culture is restricted to large, automated hot‐houses. This limits the ability of this technology to be scaled rapidly. With significant active research in this area, plant expression could yet be a future technology for large‐scale commercial production of mAbs.

Aglycosylated mAbs and antibody fragments can be produced in *Escherichia coli* both by periplasmic and cytosolic expression.^67^ This is attractive because *E. coli* can be cultured rapidly and attain high expression levels. However, *E. coli* does not have glycosylation machinery so if glycosylation is important for activity, this can be a significant limitation. Currently, *E. coli* has been employed primarily as an expression system to help screen for mAbs and only employed for clinical production of antibody fragments.^68^

Yeast expression systems have been used for clinical production of mAbs. In particular, *Saccharomyces cerevisae* has been used for expressing commercial biotherapeutics.^69^ However, a key limitation has been the generation of excessive non‐mammalian glycosylation patterns in Saccharomyces. Additionally, expression levels of full length mAbs in Saccharomyces has been limited owing to misfolding in the endoplasmic reticulum and trafficking. Pichia pastoris is emerging as a better system for recombinant protein expression. This is a methylotrophic yeast that can be cultivated at very high cell densities. Promoters used in Pichia systems are very strong and result in significant expression levels (up to 20 g/L) along with extracellular secretion. Glycosylation in Pichia is less extensive than in Saccharomyces. Engineered strains of Pichia have eliminated issues with protease expression and have also limited the generation of highly mannosylated glycoforms. One remaining challenge for this system is the paucity of chaperones for appropriate protein folding in this expression system. As a result, the product can exist in multiple conformations. However, as engineered strains of Pichia are developed, this hurdle can be overcome. High productivity in Pichia could make this an attractive future candidate for mAb expression.^70^, ^71^

Another emerging platform for biopharmaceutical production is that of microalgae production systems.^72^ Microalgae are photosynthetic microorganisms that have been cultured in very large volume fermenters. Microalgae have been employed for production of industrial biotechnology products. At this point of time, microalgae fermentation systems are still relatively low yielding. Additional hurdles including glycosylation and other post‐translational modifications will also need to be overcome before this expression system finds acceptance for biopharmaceutical production.

## 5 Conclusions

This chapter has discussed the need for a platform approach for mAbs and its utility in accelerating the progression of many different therapeutics toward the clinic and market. The use of a platform approach has enabled many biopharmaceutical companies to successfully progress mAbs from gene to IND in a year or less. Based upon their internal antibody construct, cell line and cell culture process each biopharmaceutical organization has developed its favorite platform approach. Latest trends include the use of multimodal chromatography as part of the process platform and the use of two high loading polishing steps in a flow‐through mode of operation. These modifications have enabled even broader applicability of the mAb platform as well as are meaningfully addressing the throughput bottleneck in downstream processing.

As cell culture productivity continues to advance, other alternative formats to help improve the productivity of the downstream process are being advanced. These include the operation of the Protein A chromatographic step in a continuous mode rather than a batch format. Continuous processing could conceivably be extended for the entire downstream process in the future. Nonchromatographic separation steps using precipitation or ATPS are another possible future direction for mAb downstream processing. The next decade will see further evolution of the mAb downstream process platform based on the drivers of productivity and new molecule formats.
